# Utility of the rurality index for Japan for exploring good practice solutions for declining birthrates in rural areas

**DOI:** 10.1002/jgf2.714

**Published:** 2024-07-04

**Authors:** Osamu Nomura, Yuki Soma, Makoto Kaneko

**Affiliations:** ^1^ Department of Health Sciences Education Hirosaki University Hirosaki Japan; ^2^ Faculty of Education Hirosaki University Hirosaki Japan; ^3^ Department of Health Data Science Yokohama City University Yokohama Japan

**Keywords:** birth rate, rural area, rurality index for Japan

## Abstract

**Background:**

Japan is experiencing unprecedented decreasing birthrates. This preliminary study aimed to identify the municipalities in Aomori Prefecture that are successfully tacking declining birthrates using the Rurality Index for Japan (RIJ).

**Methods:**

We obtained 100‐level RIJ and census data from 1980 to 2020 for children (age < 15 years) in the municipal population of Aomori Prefecture.

**Results:**

The analysis revealed generally weak but significant relationships between population variables and the RIJ.

**Conclusions:**

The RIJ is effective for identifying good practice communities that have developed and implemented solutions to prevent declining birthrates at the community level.

## Graphical abstract

The child population in Aomori Prefecture has been declining over the past decade, and this decline is statistically related to rurality, as indicated by the Rurality Index for Japan (RIJ). By visualizing the relationship between the child population and the RIJ across all municipalities in Aomori Prefecture, we identified the “excellent outlier” municipality that have a high percentage of child population despite having a high RIJ.

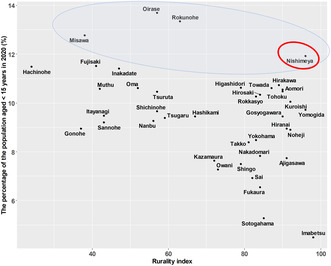

## 1 INTRODUCTION

Japan is facing a population crisis resulting from a declining birthrate, which may lead to a decline in social functions in the country. Rural areas are more vulnerable to this problem than are urban areas because rural areas have fewer social resources, such as financial and human capital, to deal with a low birthrate. Consequently, this problem can lead to a decline in the quality of local health care and community activities. Aomori Prefecture, located in the northern Tohoku region, is a representative prefecture where rurality is assumed to be related to ruralness. In 2022, the annual number of births in the prefecture was 5985 (down 8.1% from the previous year), and the birthrate was 1.24, both the lowest in statistical history.

In a validation study on the Rurality Index for Japan (RIJ), which aims to develop objective measures of rurality to improve rural health care in Japan, experts in rural medicine concluded that the elements of rurality are aggregated into low population density, long distance to the nearest secondary or tertiary hospital, and residence on remote islands or in special areas with heavy snow.^1^ The population, healthcare system, and climatic conditions of the municipalities in Aomori Prefecture resulted in high RIJ scores in that study, indicating “high” rurality in the prefecture. Although low birthrates and rurality are closely related and could be statistically correlated, this relationship may not necessarily apply to all municipalities in the prefecture. It is still possible for some municipalities located in rural areas to have high birth rates. These outliers from the correlation between birthrates and rurality can provide some hints for the design of more effective healthcare policies to increase the birthrate. The good practice policies applied in these municipalities to improve the birthrate can be a model initiative to prevent or reduce the declining birthrates seen in other areas of Aomori Prefecture and Japan. Given this background, the present preliminary study aimed to identify outlier municipalities in Aomori Prefecture that have a relatively high birthrate despite their rurality, as indicated by the RIJ.

## 2 METHODS

### 2.1 Study design

This ecological study aimed to examine the relationship between the population of children (aged < 15 years) and RIJ scores in Aomori Prefecture in the northern Tohoku region of Japan.

### 2.2 Measurements

Local government administrative boundaries in Aomori Prefecture were obtained from the National Land Numerical Information Download Service.^2^ Census data in 5‐year increments from 1980 to 2020 regarding the municipal population aged <15 years were obtained from e‐stat.^3^ The percentage of the population aged <15 years in each municipality in Aomori Prefecture and the rate of population decline of this population from the base year of 1980 were described by municipality according to administrative boundaries as of the year 2020. The rate of population decline was calculated using the following formula:

$$\begin{array}{c} \text{Rate of population decline for those aged}<15\;\text{years in each year}=\\ \frac{\text{Population aged}<15\;\text{years in}\;1980-\text{Population aged}<15\;\text{years in each year}}{\text{Population aged}<15\;\text{years in}\;1980} \end{array}$$

The RIJ by zip code and municipality was used to assess the degree of rurality of each area.^1^ These 100‐level data were obtained from the RIJ website.^4^

### 2.3 Statistical analysis

The distribution of continuous variables was assessed using the median and first and third quartiles. Spearman's rank correlation analysis was used to examine the relationship between the percentage of the population aged <15 years and the rate of population decline in each municipality and the RIJ, which was shown on scatterplots. To explore the municipalities that appeared to be adopting effective solutions, we focused on the top 10% with larger positive residuals than the regression lines depicted in the scatterplots. Statistical analysis was performed using R (version 4.3.2), and the level of significance was set to 5%.

## 3 RESULTS

In Aomori Prefecture, both the general population and the proportion of the population aged <15 years decreased, and the rate of decline increased over the years (Table S1). The percentage of the decline in high RIJ areas tended to be low (Figure S1), whereas the rate of decline tended to be high (Figure S2). The relationships between population variables and the RIJ were generally weak, but significant (Table 1).

**TABLE 1:** Relationship between variables for the population aged < 15 years and the Rurality Index for Japan.

	Percentage of the population aged < 15 years	Rate of decline of the population aged < 15 years
1980	−0.152	—
1985	−0.092	0.167
1990	−0.054	0.280
1995	−0.228	0.296
2000	−0.420*	0.351*
2005	−0.392*	0.347*
2010	−0.380*	0.341*
2015	−0.385*	0.330*
2020	−0.304	0.297

*Note*: *N* = 40.

* *p* < 0.05 in Spearman's rank correlation analysis.

**FIGURE 1:**
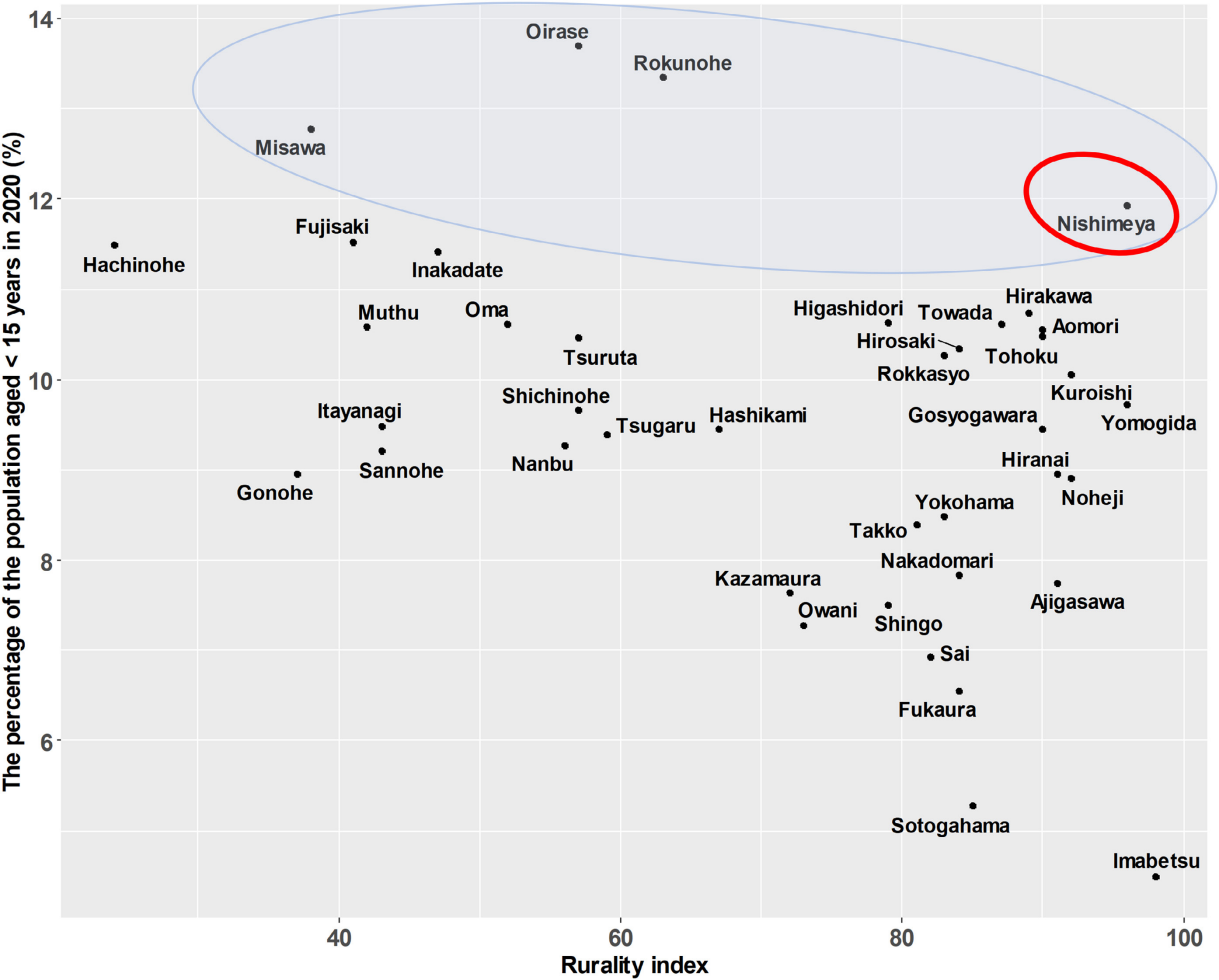
Scatterplots of the percentage of the population aged <15 years in 2020 and the Rurality Index for Japan by municipality.

Figure 1 shows scatterplots of the percentage of the population aged <15 years in 2020 by municipality and the RIJ. Four municipalities were identified in the top 10% with larger positive residuals from the scatterplot regression lines, and Nishmeya village, which had the highest RIJ score, was identified as the representative outlier.

## 4 DISCUSSION

The results of the present preliminary study indicated that the population aged <15 years in Aomori Prefecture has been declining over the past decade, and that this decline is statistically related to rurality, as indicated by the RIJ. Furthermore, by visualizing the relationship between the population aged <15 years and the RIJ across all municipalities in Aomori Prefecture, we identified an “excellent outlier” municipality that has a high percentage of the population aged <15 years despite having a high RIJ score.

According to a review of the Nishimeya village website, this municipality successfully increased this population through an initiative that intensively improved childcare services. That initiative included a free pregnancy checkup and examination program, free medical care for all children, a free vaccination program for all children, free day care for infants, and tuition assistance for high school and university students. This finding indicates that the RIJ can be used as a measurement tool to identify good practice communities that have developed and implemented solutions to help prevent declining birthrates.

The causes of declining birthrates are multifactorial and complicated; therefore, it may be impossible to identify a one‐size‐fits‐all solution to address this problem. In this sense, other communities in the same prefecture may follow and partially implement the initiatives of “excellent outlier” municipalities to help prevent declining birthrates.

### 4.1 Limitations

In this study, we only analyzed population data from Aomori Prefecture; therefore, whether our findings are applicable to other prefectures in Japan remains unclear. In addition, we did not examine local government policies and other resources on the municipality websites. Further research is needed to examine the generalizability of the present findings and overcome these limitations.

## FUNDING INFORMATION

This work was supported by JSPS KAKENHI Grant Numbers 24 K02670.

## CONFLICT OF INTEREST STATEMENT

The authors have stated explicitly that there are no conflicts of interest in connection with this article.

## ETHICS STATEMENT

Ethics approval statement: None.

Patient consent statement: None.

Clinical trial registration: None.

## Supporting information


Data S1.

## Data Availability

This study was conducted using published online data.
