# Supplementary material for: Utility of the rurality index for Japan for exploring good practice solutions for declining birthrates in rural areas
Source: J Gen Fam Med. 2024 Jul 4;25(6):395–7. doi: 10.1002/jgf2.714 (PMC11565061; doi:10.1002/jgf2.714)
Supplement: Supplementary file 1 — Data S1. [file JGF2-25-395-s001.docx]

TABLE S1 Population, percentage of the population, and rate of decline of the population aged < 15 years in Aomori Prefecture

|  |  | Population aged < 15 years | | | Percentage of the population  aged < 15 years (%) | | | Rate of decline of the population  aged < 15 years (%) | | | |
| --- | --- | --- | --- | --- | --- | --- | --- | --- | --- | --- | --- |
|  |  | Median | 1st quantile | 3rd quantile | Median | 1st quantile | 3rd quantile | Median | 1st quantile | 3rd quantile |  |
| 1980 |  | 3988 | 2435 | 6512 | 23.7 | 22.8 | 25.2 | - | - | - |  |
| 1985 |  | 3592 | 2194 | 5911 | 22 | 21 | 23.2 | 9.33 | 6.3 | 12.2 |  |
| 1990 |  | 3068 | 1788 | 5060 | 19.5 | 18.4 | 20.3 | 24.7 | 19.9 | 29.9 |  |
| 1995 |  | 2588 | 1449 | 4509 | 16.5 | 16 | 17.5 | 36.3 | 30.6 | 42.9 |  |
| 2000 |  | 2184 | 1160 | 4446 | 14.6 | 13.8 | 15.1 | 45.6 | 38.9 | 53.7 |  |
| 2005 |  | 1799 | 998 | 4262 | 12.9 | 11.8 | 13.7 | 53.8 | 45.7 | 62.9 |  |
| 2010 |  | 1532 | 833 | 3878 | 11.5 | 10.2 | 12.5 | 61.2 | 52.4 | 69.1 |  |
| 2015 |  | 1376 | 658 | 3447 | 10.6 | 9.25 | 11.5 | 68.7 | 58.8 | 75.1 |  |
| 2020 |  | 1154 | 496 | 2981 | 9.57 | 8.47 | 10.6 | 72.9 | 61.9 | 79.9 |  |
|  |  |  |  |  |  |  |  |  |  |  |  |
| RIJ* (1-100) |  | 80 | 56.8 | 89.2 |  |  |  |  |  |  |  |

* Rurality Index for Japan


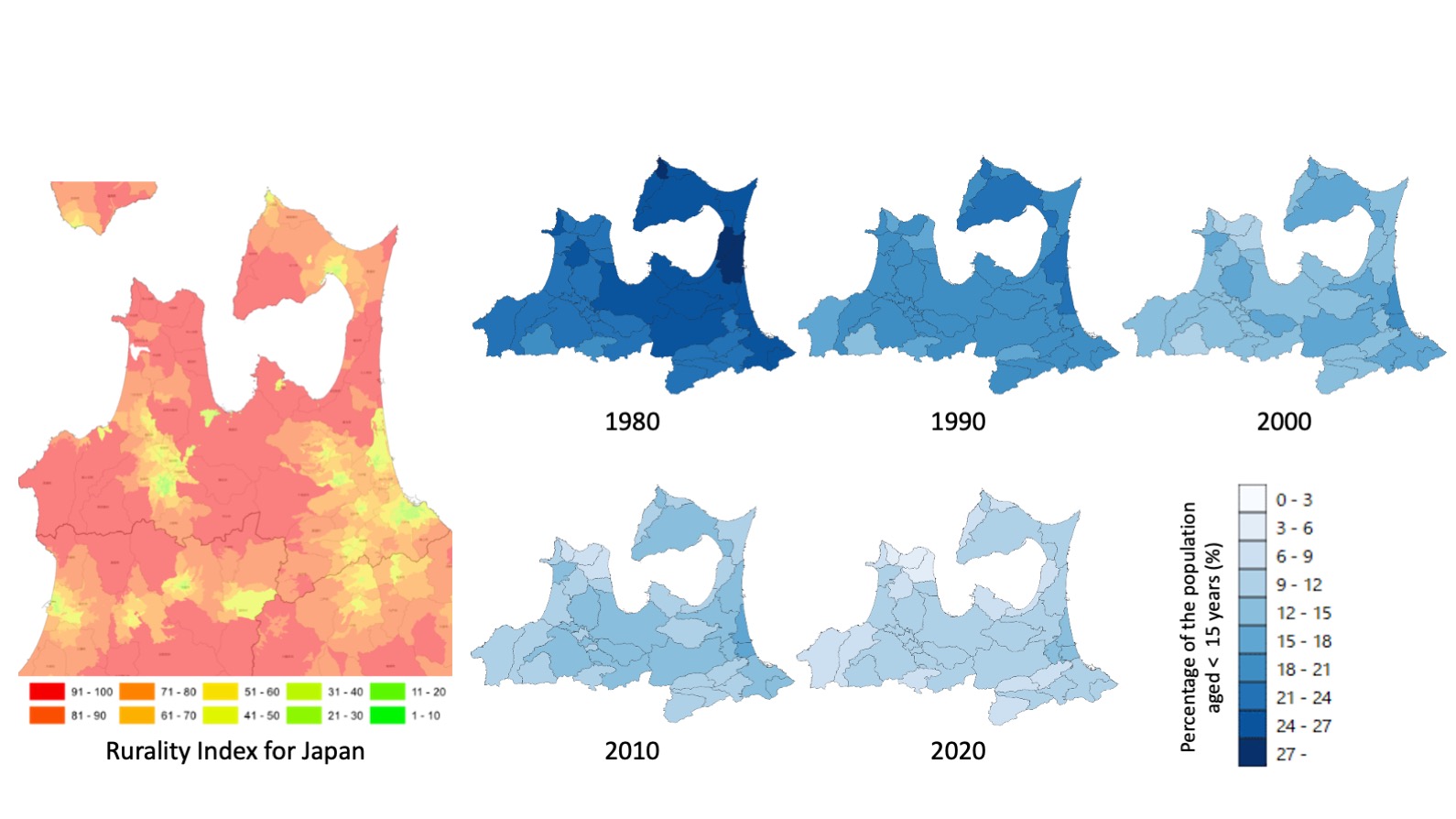


FIGURE S1 Percentage of the population aged < 15 years in 5-year increments and the Rurality Index for Japan


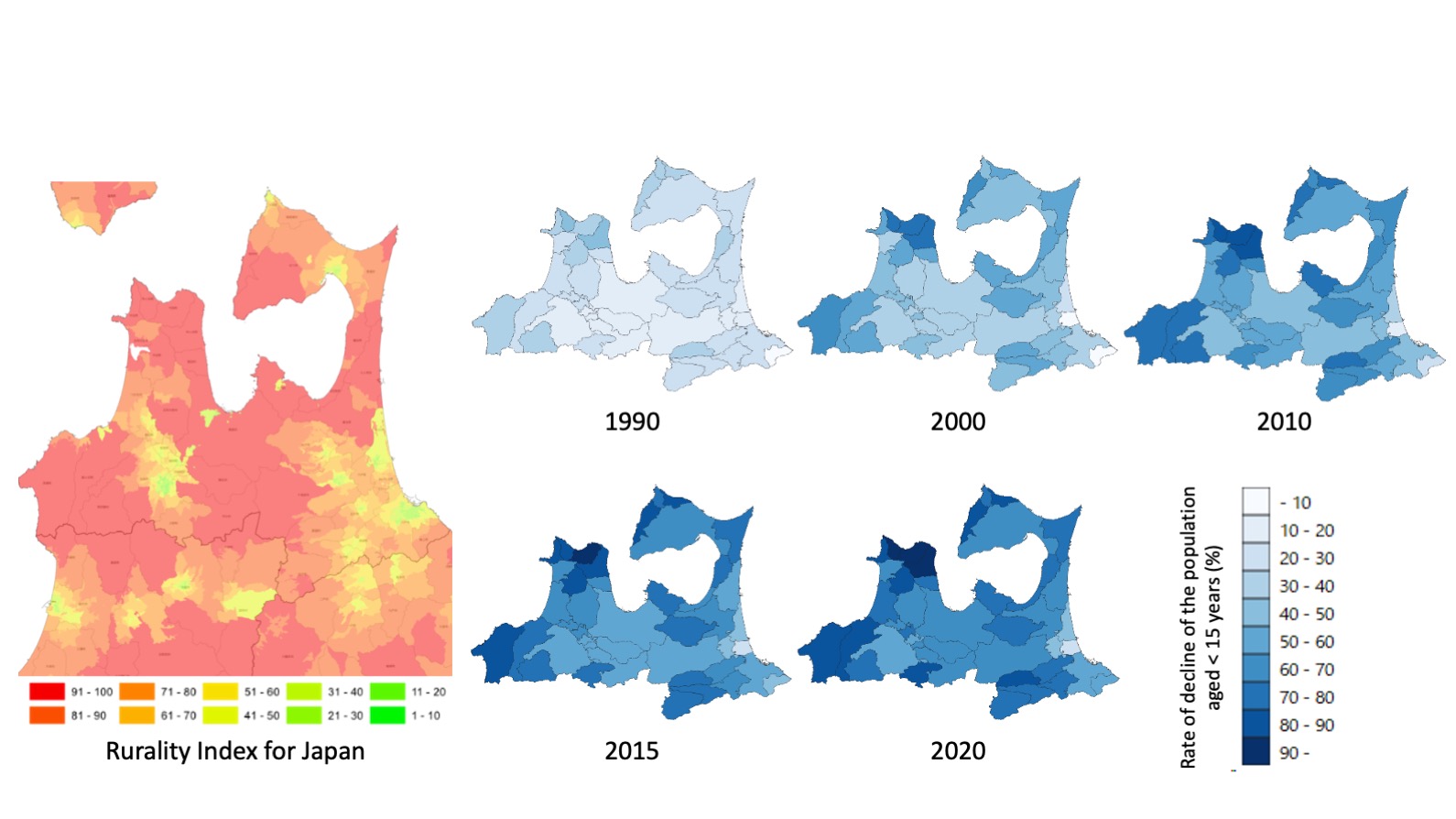


FIGURE S2 Rate of decline of the population aged < 15 years in in 5-year increments and the Rurality Index for Japan
